# A nondestructive method to estimate the chlorophyll content of *Arabidopsis* seedlings

**DOI:** 10.1186/s13007-017-0174-6

**Published:** 2017-04-14

**Authors:** Ying Liang, Daisuke Urano, Kang-Ling Liao, Tyson L. Hedrick, Yajun Gao, Alan M. Jones

**Affiliations:** 1 Department of Biology, The University of North Carolina at Chapel Hill, Coker Hall, CB#3280, Chapel Hill, NC 27599-3280 USA; 2 College of Natural Resources and Environment, Northwest A&F University, Yangling, 712100 Shaanxi China; 3 Department of Pharmacology, University of North Carolina at Chapel Hill, Chapel Hill, NC 27599-3280 USA

**Keywords:** *Arabidopsis thaliana*, C/N sensing, Chlorophyll content, ColorChecker chart, Heterotrimeric G protein complex, Stress assay

## Abstract

**Background:**

Chlorophyll content decreases in plants under stress conditions, therefore it is used commonly as an indicator of plant health. *Arabidopsis thaliana* offers a convenient and fast way to test physiological phenotypes of mutations and treatments. However, chlorophyll measurements with conventional solvent extraction are not applicable to *Arabidopsis* leaves due to their small size, especially when grown on culture dishes.

**Results:**

We provide a nondestructive method for chlorophyll measurement whereby the red, green and blue (RGB) values of a color leaf image is used to estimate the chlorophyll content from *Arabidopsis* leaves. The method accommodates different profiles of digital cameras by incorporating the ColorChecker chart to make the digital negative profiles, to adjust the white balance, and to calibrate the exposure rate differences caused by the environment so that this method is applicable in any environment. We chose an exponential function model to estimate chlorophyll content from the RGB values, and fitted the model parameters with physical measurements of chlorophyll contents. As proof of utility, this method was used to estimate chlorophyll content of G protein mutants grown on different sugar to nitrogen ratios.

**Conclusion:**

This method is a simple, fast, inexpensive, and nondestructive estimation of chlorophyll content of *Arabidopsis* seedlings. This method lead to the discovery that G proteins are important in sensing the C/N balance to control chlorophyll content in *Arabidopsis*.

**Electronic supplementary material:**

The online version of this article (doi:10.1186/s13007-017-0174-6) contains supplementary material, which is available to authorized users.

## Background

The chlorophyll content of leaves is an indirect indicator of the health and nutritional status of the plant [1]. Traditional methods to calculate the chlorophyll content include a destructive chemical extraction and a non-destructive measurement of chlorophyll fluorescence. The former method, while direct, is tedious and unsuitable for continuous monitoring individual plants because of its destructive manner. The latter method needs expensive instruments of which none are presently suitable for small leaves such as the commonly used *Arabidopsis* cotyledons. It is important to develop a non-destructive method to estimate chlorophyll content for *Arabidopsis* because it is a genetic model plant, however traditional chlorophyll extraction is not useful due to the small size of the *Arabidopsis* leaves grown on agar plates. Recently, digital photographic imaging showed great promise for quantitating plant phenotypes [2]. Indirect methods are available but none are yet suitable for *Arabidopsis*. Sass et al. [3] developed a protocol to convert the RGB values of a color image into a hue saturation value (HSV), and showed that the hue value was correlated to the chlorophyll content estimated by a destructive method. A similar color-image method was used to assess the nitrogen status of rice under natural light condition [4]. Riccardi et al. [5] found that an exponential function model displays the best correlation between the RGB values and the chlorophyll content through single and multiple regression in quinoa and amaranth leaves. No similar color-image methods have been adapted for *Arabidopsis* chlorophyll content, in particular *Arabidopsis* seedlings grown on agar plates. The lack of a quantitative method for measuring chlorophyll of plate-grown *Arabidopsis* restricted previous studies on stress-induced phenotypes to subjective assessment without quantitation [6, 7].

Chlorophyll content in leaves is affected by the carbon (C) and nitrogen (N) balance. Genetics studies using *Arabidopsis* revealed that the C/N balance is regulated through multiple signaling cascades of *abscisic acid*-*insensitive 1* (*ABI1*), *hexokinase 1* (*HXK1*), nitrate transporters, glutamate receptor (*AtGLR1.1*) and heterotrimeric G proteins [6, 8–12]. Many mutant alleles in these pathways confer altered chlorophyll content regulated by the C/N balance in *Arabidopsis* [6, 9, 12], therefore these mutants are useful for validating the utility of our digital image method. Heterotrimeric G proteins consist of one canonical Gα subunit (*GPA1*), one Gβ subunit (*AGB1*), three Gγ subunits (*AGGs*) and three atypical extra-large Gα proteins (*XLGs*) [13–15] in *Arabidopsis*. G protein signaling pathway senses glucose levels in the environment [16, 17], and is also involved in nitrogen use efficiency in rice [10]. *Regulator of G protein Signaling* 1 protein (AtRGS1) is a component of the glucose sensor [11, 12, 18]. This protein modulates the activation state of G signaling.

In this study, we describe a convenient and nondestructive method to estimate leaf chlorophyll of *Arabidopsis* seedlings grown on agar plates using calibrated-RGB images. We also provide instructions how to adapt it to other small leave samples. We quantitated chlorophyll content in small *Arabidopsis* seedlings grown on different C: N ratios in the agar medium. The results indicated that G proteins play important roles in sensing and/or responding to the C/N balance in this chlorophyll response.

## Materials needed

1. *Arabidopsis* seedlings grown on agar plates. In this study, we used the following T-DNA insertion mutant alleles: *agb1*-*2* [19], *rgs1*-*2* [20], *gpa1*-*3* [21], *xlg1xlg2xlg3* [13] which combines these alleles *xlg1*-*1* (SAIL_760H08) [13], *xlg2*-*2* (SALK_062645), *xlg3*-*2* (SAIL_107656) [13], and *xlg/gpa1* which combines the *xlg1*-*1*, *xlg2*-*1*, *xlg3*-*2* and *gpa1*-*3 alleles above* [22] (in press).
2. A digital camera that captures images in RAW format.
3. X-rite ColorChecker classic chart (http://xritephoto.com/colorchecker-classic).
4. Software: ImageJ https://imagej.nih.gov/ij/(public source; imageJ 1.50i is recommended),
  DNG converter http://www.adobe.com (public source),
  DNG profile editor http://www.adobe.com (public source) and
  PhotoShop or Lightroom http://www.adobe.com (license required).
  Optional software: Matlab http://www.mathworks.com/(license required).
5. Plug-in programs for ImageJ are provided here in Additional file 1: S1, Additional file 2: S2, Additional file 3: S3 and Additional file 4: S4. Additional file 1: S1 and Additional file 2: S2 are for chlorophyll calculation and Additional file 3: S3 and Additional file 4: S4 are for reading the RGB values of the images. Additional file 1: S1 and Additional file 3: S3 are the .java source code used to generate the .class files (Additional file 2: S2 and Additional file 4: S4). In most cases, only the .class files are needed, however the corresponding .java files are provided for those wanting to examine or improve the programs.

*Note 1:* This plugin was successfully tested in both ImageJ 1.48v and ImageJ 1.50i.

6. Optional: Materials for chlorophyll extraction: 80% acetone in water, spectrophotometer

## Plant growth

1. Grow *Arabidopsis* seedlings in the light on square 8 cm × 8 cm plastic Petri plates (VWR; Cat No. 60872-310) with a 40-mL layer of agar and media suitable for plant growth.
2. Thirty-six seedlings are placed individually within the 36-gridded area of the plate; one seedling per grid. There should be no overlap of seedlings. This spacing is important for the software to automatically detect seedlings for chlorophyll calculations. There is also an option described later to create a different grid then the default 6 × 6 grid (step 8 of the protocol).

### *Note 2:*

It is not necessary to use a square petri dish on which to arrange the seedlings; any rectangle background with samples in a matrix format will work. It is also possible to treat seedlings in liquid culture or on some matrix other than agar and then transfer them to the square agar plates for photography.

### *Note 3:*

For this study, seedlings were grown on the indicated media arranged on square plates as described above and photographed as will be described below. Specifically, Murashige and Skoog (MS) Modified Medium w/o Nitrogen (Plantmedia; Cat No. 30630200-1) supplied with 0.8% phytoagar and 1 g/L MES was used. The pH was adjusted to 5.7 with KOH. Filter-sterilized d-glucose was added to the medium to adjust the glucose concentration as indicated. A stock solution of 1 M KNO_3_ was used as the nitrogen resource. The agar plates of sterilized seed, sealed with a gas-permeable tape, were stratified at 4 °C for 3–5 days in the dark. The plates were placed horizontally under constant dim light (35–50 µEm^−2^ s^−1^) at 23 °C for 12 days. Images were obtained on the 12th day. Plants were also grown on soil in a long day chamber (200 µEm^−2^ s^−1^, 16 h light/8 h dark) at 23 °C for 4–8 weeks as indicated in the experimental description.

## Protocol

**Fig. 1:**
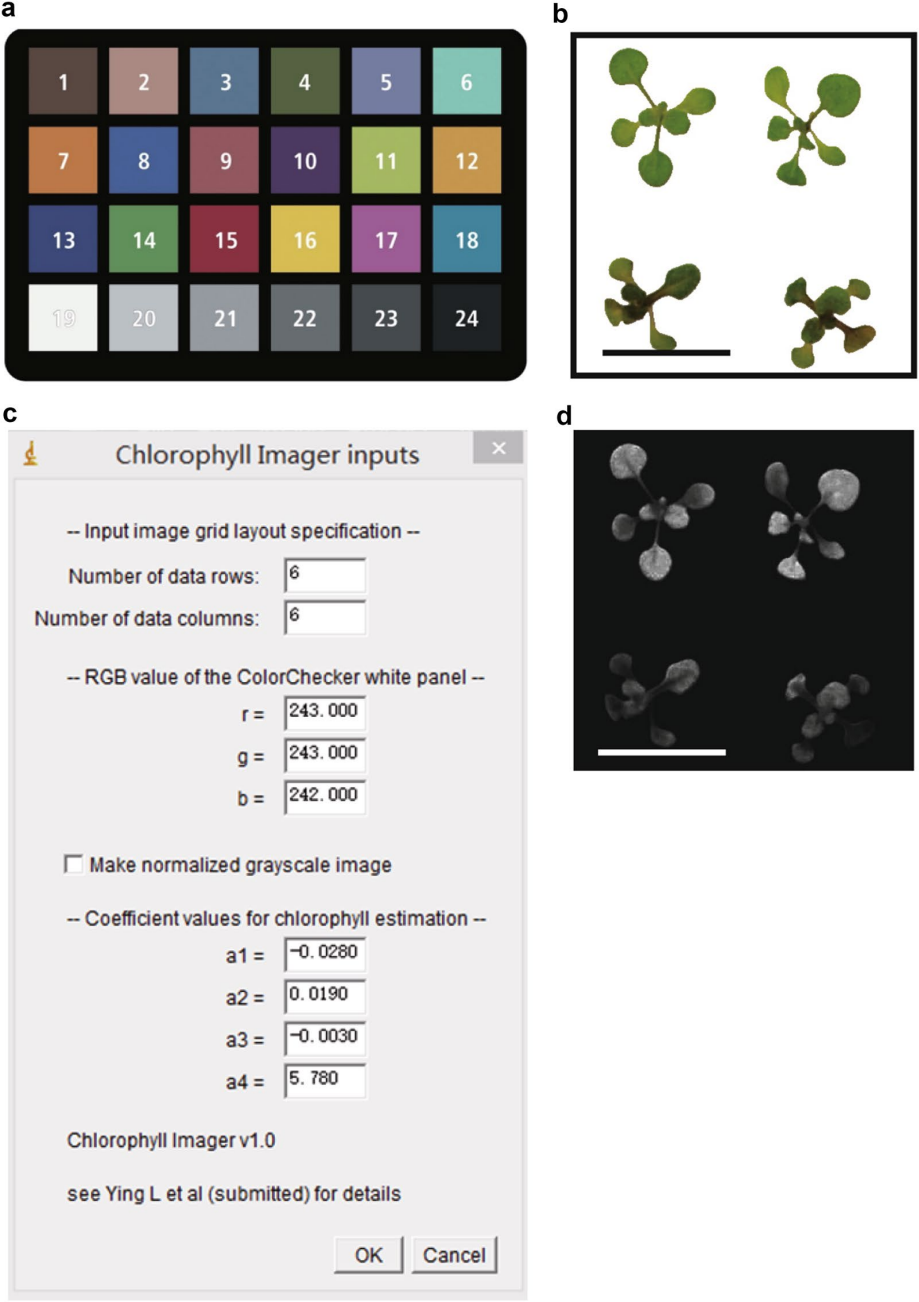
Examples of images needed in converting RGB value to chlorophyll content. **a** The *picture* of the X-rite ColorChecker classic chart. **b** The original picture of the *Arabidopsis* seedlings grown on the plates under 4% glucose and 2 mM nitrogen for 12 days. **c** The dialogue box of the plugin in ImageJ. **d** The *grey scale pictures* represent the chlorophyll content, which is calculated by the equation estimate the chlorophyll content

1. Photograph seedlings arranged on the square plates and save as RAW files. Any digital camera that stores images as RAW files can be used.
*Note 4*: For this study, an Olympus digital camera E-3 captured the RGB color images and the images stored as ORF file, a type of RAW format. Settings for the digital camera were the following: aperture = f/7, shutter speed = 1/100 s, ISO = 400, quality = F, file storage = RAW. Shutter speed should be adjusted according to the actual ambient light condition.
*Note 5*: Acquire images of the X-rite ColorChecker classic chart both immediately before and after acquiring images of the samples (Fig. 1a). The ColorChecker chart is to make sure the final data comparable despite different light conditions and cameras but may not be necessary. Before and after images of the chart are acquired to test whether the light condition is consistent during the photographing. Check the RGB values of the X-rite Colorchecker chart boards as described below. Should you find that the starting and ending values are different, it will be necessary to stabilize the light environment and re-acquire sample images. If the light condition is stable in the lab, it is not necessary to acquire two images every time. It is important to use the same settings and the same light conditions for all the images to be compared.
*Note 6*: The image size for the plate is the same as for the ColorChecker chart, approximately 210 × 300 mm. The square plate is placed in the center of this field for imaging.
*Note 7*: The light should be uniformly distributed. Tests for position effect in this field were determined to be a maximum of 4.8% (n = 36) (Additional file 5: S5) of the chlorophyll values. This value was calculated by estimating the chlorophyll content of 36 individual seedlings on a plate placed in the center and 4 corners of the field.
2. Convert the RAW files to DNG format using the DNG converter. There are many formats of RAW file; one is a DNG. If your camera stores the files in DNG format, nothing more is needed at this step.
3. Generate a DNG profile of the X-rite ColorChecker chart. This format can be used by Adobe PhotoShop and Lightroom programs. Launch the DNG profile editor, and then click on the ‘Chart’ tab. Then load the DNG file checkerchart. Use the mouse to position the four colored circles in the image at the centers of the four corner color panels of the chart. The colors of the circles should correspond to the colors of the patches. Leave the popup menu set at ‘Both Color Tables’ and click ‘Create Color table’ button. Then export the profile by selecting Export Profile in the File menu and name the profile as ColorCheckerChart.dcp. Make sure the file is saved in the CameraProfiles directory (default).
4. Using the Checker.dcp files generated in step 3, calibrate all seedling images using the DNG profile through the software Lightroom or Photoshop. These programs provide a step-by-step instructions for calibration. After the adjustment, export the images in jpg format labeled accordingly: IMAGE_ID.jpg.
5. Open the IMAGE_ID.jpg files in Adobe Photoshop. Clean or erase the background of the images with the eraser or magic wand/delete tools (Fig. 1b). Label files IMAGE_ID_cleaned.JPG The plugin will automatically remove slightly gray or otherwise imperfect backgrounds, but use of this capability should be carefully validated against test images with manually cleaned backgrounds.
*Note 8*: Cleaning the background is an important step. Assure that shadows are completely eliminated and only leaves remain in the image. An example of a perfectly cleaned image is provided as Additional file 6: S6.
6. Open ImageJ and install the plugin Chloropyll_Imager provided in the Additional file 2: S2. Additional file 1: S1 is the .java code which could also be used if preferred.
7. Open ColorChecker.jpg in ImageJ and record the RGB values designated r, g, b of the white background. The r, g, and b values are obtained using the plugin labeled ‘RGB_measure provided in Additional file 4: S4. Additional file 3: S3 is the .java code which could also be used if preferred. Select the white panel of the ColorChecker chart and run the plug-in. The corresponding r, g, b values of the white panel will appear in a table.
8. Open IMAGE_ID_cleaned.jpg files in ImageJ and run the plugin “Chorophyll_Image” which in step 6 you had saved in the ImageJ ‘plugins folder. A dialog box will appear asking for the number of rows and columns (Fig. 1c). Enter these values or use the default value of 6 rows × 6 columns. This step divides the images into 36 parts with 6 rows × 6 columns. The dialog box also will request the RGB values of the white panel recorded in step 7. Enter these values. By clicking “OK”, you will generate a table of the chlorophyll content as ng/mm^2^. The dialogue also asks if you want to make a normalized grayscale image (Fig. 1d). If so, click the box and a grey scale image will also be presented as output (see Additional file 7: S7 for an example). This greyscale image offer a spatial map of chlorophyll on a leaf; i.e. 2-dimensional information is provided.

*Note 9*: The default coefficient values for chlorophyll estimation are shown as a1, a2, a3, and a4 in the boxes of the plug-in menu. These values can be changed to fit other types of samples such as leaf pieces. Other coefficients can be determined as described in the “Do it yourself” section below.

9. Validation using a test sample. A cleaned image with 6 rows × 6 columns seedlings is provided as Additional file 6: S6. The RGB values of the white background for this figure are 171.666, 171.297, and 171.256, respectively. After running the plugin, you will obtain the chlorophyll content of the 36 seedlings provided in the test file. In order to confirm correct operation of the program, compare your calculated results with the values shown in Additional file 8: S8.

## Chlorophyll extraction

For validation purposes, we compared our method to extracted chlorophyll. Chlorophyll content was estimated by spectrophotometry of samples prepared by 80% acetone extraction. The leaves were incubated at room temperature in a 1.5-mL tube with 1 mL 80% acetone solution for at least 24 h then clarified by centrifugation for 5 min at 15,000*g*. In this study, absorbance of the supernatant was measured at wavelengths 645, 646, and 663 nm (A_645_, A_646_, and A_663_) with a Shimadzu UV-3000™ dual-wavelength, double-beam spectrophotometer, although any spectrophotometer is suitable. Complete spectra were taken during development of this protocol in order to assure that the predominant absorbance was from chlorophyll; this is not routinely necessary. Samples having absorbance greater than 1 were diluted by half with 80% acetone and re-evaluated. Chlorophyll concentration was estimated following the Lichtenthaler’s equations (A) [23] and the Arnon’s equations (B) [24] as follows:

A. 1 $$ \begin{aligned} & {\text{Chlorophyll}}\,{\text{a}}\, (\upmu{\text{g/mL)}} = - 1.93\,A_{646} + 11.93\,A_{663} \\ & {\text{Chlorophyll}}\,{\text{b}}\,(\upmu{\text{g/mL}}) = 20.36\,A_{646} - 5.50\,A_{663} \\ & {\text{Total}}\,{\text{chlorophyll}}\, (\upmu{\text{g/mL)}} = 6.43\,A_{663} + 18.43\,A_{646} \\ \end{aligned} $$
B. 2 $$ \begin{aligned} & {\text{Chlorophyll}}\,{\text{a}}\, (\upmu{\text{g/mL)}} = 12.7\,(A_{663} ) - 2.69\,(A_{645} ) \\ & {\text{Chlorophyll}}\,{\text{b}}\, (\upmu{\text{g/mL)}} = 22.9\,(A_{645} ) - 4.68\,(A_{663} ) \\ & {\text{Total}}\,{\text{chlorophyll}}\,(\upmu{\text{g/mL}}) = 20.2\,(A_{645} ) + 8.02\,(A_{663} ) \\ \end{aligned} $$

The area of the leaves were measured by software Image J and chlorophyll content and the total chlorophyll content per leaf area was expressed as ng/mm^2^.

## Fitting parameters for the function and “Do it yourself” validation for other types of chlorophyll containing samples

The default coefficient values used above are described here in the event that the user needs to modify this tool to obtain different coefficients for other types of chlorophyll samples such as leaf pieces. A least squares method was used to search for the coefficients for the exponential function equation to estimate chlorophyll contents from RGB values. If the user desires to validate this method on their own using their own images and chlorophyll samples, then follow these steps:

1. Read the RGB values of each sample. A plugin labeled ‘RGB_measure.java’ is provided in Additional file 4: S4.
2. Measure the chlorophyll content using chemical extraction (see “Chlorophyll extract” section). The chlorophyll content should be expressed as total chlorophyll per leaf area, ng/mm^2^.
3. Combine the datasets of R, G and B values obtained from step 1 with the chlorophyll content estimated by chemical extraction from step 2 to search for the coefficient with a least-squares method. An excel file, Additional file 9: S9, labeled EXAMPLE.xlsx, provides a sample dataset of RGB values with chlorophyll content data. Replace your datasets with the original ones. This spreadsheet provides the input chlorophyll and color data used to fit the best coefficients.
4. Open Matlab and upload the appropriate ‘Leastsquareequation’ script provided in Matlab code (Additional file 10: S10). The provided code finds the best fit of the coefficients using the following equation
  3 $$ Chl = EXP\left( {a1*R*r/243 + a2*G*g/243 + a3*B*b/242 + a4} \right) $$
  where R, G and B refer to the color of the plants read from the plug-in described above, and r, g and b refer to the corresponding RGB values of the white background. Before running the scripts, change the input of r, g and b to these new values.
  Having the new coefficients enables you to estimate chlorophyll in other samples nondestructively.

### *Note 10:*

For Matlab, paste the provided MatLab script ‘Leastsquareequation.m’ (Additional file 10: S10) and the EXAMPLE.xlsx (Additional file 9: S9) into the same folder.

### *Note 11:*

Other color charts may be used but the values of white may be different than for the X-rite ColorChart. In this case, replace the values 243, 243, 242, respectively in Eq. (3) with the corresponding white background values.

## Results and discussion

### From RGB value to chlorophyll content

We adapted the method of Riccardi et al. [5] to *Arabidopsis* seedlings grown on culture plates and compared this method to biochemical extraction of chlorophyll. We extracted chlorophylls with acetone, measured absorbance spectra, then calculated chlorophyll content in the solvent extract using both the Lichtenthaler’s [23] and Arnon’s equations [24] (Eqs. 1, 2).

**Fig. 2:**
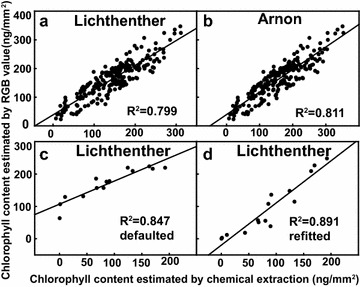
Correlation of the chlorophyll content estimated by RGB value and chemical extraction. **a**, **b** Comparison of extracted chlorophyll calculated by the Lichthenther (**a**) and Arnon (**b**) methods to chlorophyll content estimated by RGB value of *Arabidopsis* seedlings grown in agar plates under different C/N treatment (n = 234, four independent experiment). **c**, **d** Chlorophyll content estimated by RGB value and chemical extraction in soil-grown-plant using the defaulted coefficients (**c**) versus the refitted coefficients (**d**), respectively (n = 15)

Samples from 12-day-old *Arabidopsis* shoots from seedlings grown under different C/N treatments on square plates as described under plant growth were used to search for the parameters for the exponential function model (see Additional file 11: S11). To assure that the images taken by different cameras are comparable, we incorporated a standard to enable comparison of published data. The X-rite ColorChecker chart (Fig. 1a) was used to make a DNG profile and to adjust the white balance. Another critical variable to account for is the light intensity and color in the room, chamber, greenhouse or field. The X-rite ColorChecker chart solves these problems and makes the assay applicable to artificial and natural light. The software Image J was used to measure average R, G and B values of individual leaves. We used the exponential function model

4 $$ Chl_{i} = e^{{a_{1} r_{i} + a_{2} g_{i} + a_{3} b_{i} + a_{4} }} $$

to estimate the chlorophyll content where the (*r*_*i*_, *g*_*i*_, *b*_*i*_) represents the R, G or B value for each sample where i accounts for the sample index [5]. We used a biochemical extraction with Lichtenthaler’s (Eq. 1) equations to measure the chlorophyll content and fitted coefficients for the equation. We took samples from 4 independent experiments (n = 234 samples; Additional file 11: S11) and determined the coefficients: *a*_1_ through *a*_4_ in the equation (Eq. 4) using the least squares method in the MATLAB environment (Fig. 2a, b).

### Robustness of the default parameters

Our method is optimized for *Arabidopsis* seedlings grown on agar plates, a common format for *Arabidopsis* researchers. Since the default parameter values were generated using chlorophyll extracted seedlings grown under one light condition, a concern is how well these values apply to other growth conditions. To test this, we compared chlorophyll estimation in two extreme growth conditions. In our lab, the thickness of 4–7 week-old leaves grown on soil under a long-day condition is almost twice as seedling leaves grown on plates under constant low light (176 vs. 100 µm, respectively). We compared chlorophyll estimation of these thicker leaf pieces using the default parameters to refitted parameters. The thicker leaf pieces were imaged as described above and the extracted chlorophyll used to refit the data to generate new parameters:

$$ a_{1} = - 0.2032,\quad a_{2} = 0.115409,\quad a_{3} = 0.044964,\quad a_{4} = 8.048844. $$

The optimized parameters increased the *R* ^2^ correlation coefficient only from 0.85 to 0.89 (cf. Fig. 2c, d). This indicates that the default values are robust with regard to different growth conditions that may affect leaf thickness. Nonetheless, when extreme accuracy is required, we recommend calculating the parameters by the “Do it yourself” fitting method described above.

### Proof of utility: chlorophyll content of *Arabidopsis* seedlings grown under different C/N ratios

**Fig. 3:**
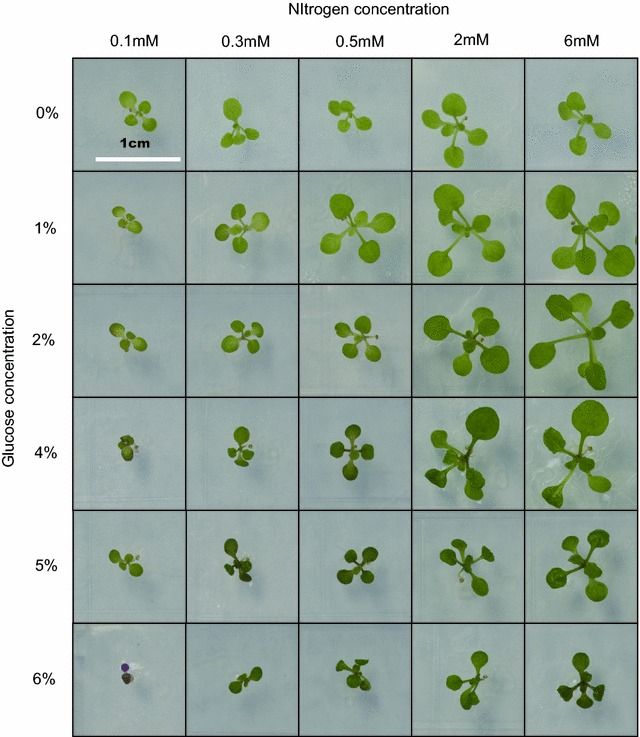
Growth of the wild type *Arabidopsis* in response to C/N ratios. The image of the plate-grown plants under different C/N ratios, including six different glucose concentration (0, 1, 2, 4, 5, and 6%) and five nitrogen (0.1, 0.3, 0.5, 2 and 6 mM KNO_3_) concentrations

In order to determine how plants respond to different C/N ratios, we tested six different glucose concentrations (0, 1, 2, 4, 5 and 6% d-glucose) and five nitrogen concentrations (0.1, 0.3, 0.5, 2 and 6 mM KNO_3_) in a matrix format. Figure 3 shows plant growth under these different C/N ratios and Table 1 shows the plants area in response to different C/N ratios. Plant area did not change in response to nitrogen under 0% glucose, although the average leaf area changed. As quantitated in Additional file 12: S12A, plant area at 0% glucose varied greatly but was statistically unchanged. When the medium contains glucose, plant area increased with increasing nitrogen. The optimal glucose concentration at the highest nitrogen concentration at 3 mM was 1–2% (Additional file 12: S12 panel A).

**Table 1:** Plant size (mm^2^) in response to glucose and nitrogen treatment

Glucose (w/v) (%)	Nitrogen concentration (mM)				
	0.1	0.3	0.5	2	6
0	8.94 a A	7.00 a A	13.59 a B	16.70 a DE	8.62 a D
1	5.93 d C	16.29 c AB	23.32 bc A	30.58 b BC	59.27 a A
2	6.74 d B	13.28 cd AB	21.20 c A	45.80 b A	55.73 a A
4	6.03 b B	9.02 b B	13.13 b B	31.85 a B	42.36 a B
5	6.75 b B	9.70 b AB	9.26 b B	22.32 a CD	23.60 a C
6	2.51 d D	6.20 cd C	8.15 bc B	11.59 b E	15.87 a CD

Growth condition and treatments are as described for Fig. 3. Data analysis is performed by software SAS8.0. Single factor analysis (n = 12). Capital letters represent similarity groups (*p* > 0.05) among glucose treatment and lowercase letters represent similarity groups among nitrogen treatments (*p* > 0.05)

The chlorophyll content correlated with the nitrogen concentration in the presence of carbon supply. We estimated the chlorophyll content at different C/N ratio. As shown in Table 2, the chlorophyll content of the Col-0 seedlings grown on 0% glucose did not change with increasing nitrogen in the medium. All the plates were grown under continuous dim light (35–50 µEm^−2^ s^−1^) at 23 °C, which decreases the photosynthesis. However, even a slight amount of glucose dramatically changed this relationship. For example, in the presence of 1% glucose, the chlorophyll content increased in response to the nitrogen concentration, and slightly decreased when the nitrogen concentration was raised further. This indicates that the chlorophyll content of the leaves is slightly influenced by nitrogen concentration but highly influenced by the carbon availability. Also, the chlorophyll content increased as the glucose concentration increased; the seedlings grown with 4% or 5% d-glucose had dark green leaves. Based on plant area, optimal growth was at 1 and 2% glucose with 6 mM nitrogen. This condition did not produce the highest chlorophyll content because the seedlings were stressed. The highest concentration of 6% glucose, with 0.1 mM nitrogen, stressed seedlings further, as was apparent by the red color of leaves caused by excessive anthocyanin pigments.

**Table 2:** Chlorophyll content (ng/mm^2^) in response to glucose and nitrogen treatment

Glucose (w/v) (%)	Nitrogen concentration (mM)				
	0.1	0.3	0.5	2	6
0	49.10 a B	38.27 a C	49.93 a C	53.85 a C	42.71 a D
1	31.82 d C	81.49 c AB	112.26 ab A	120.08 a B	98.58 b C
2	37.31 d BC	70.00 c B	72.02 c BC	125.64 a AB	105.37 b BC
4	49.36 c B	84.27 b AB	89.73 b AB	134.22 a AB	140.03 a A
5	71.66 c A	96.63 b A	104.63 b A	138.28 a A	133.65 a AB
6	44.74 b BC	99.69 a A	111.85 a A	131.07 a AB	111.33 a ABC

Data analysis is performed by software SAS8.0. Single factor analysis (n = 12). Different capital letters indicated significant differences among six glucose treatment (*p* < 0.05) and different lowercase letters indicated significant differences among five nitrogen treatment (*p* < 0.05)

### Function of G protein signaling pathway in C/N sensing

In order to analyze whether G protein signaling pathway is important for C/N sensing/responsiveness, the null mutants of the G protein under various C/N condition were assayed. The leaf area differences between the mutants of *rgs1*-*2*, *gpa1*-*3* and *agb1*-*2* is not uniform under different C/N conditions (Additional file 13: S13). For example, *agb1*-2 mutants grown under limited nitrogen (0.1 mM) are slightly larger than the wild type under 1 and 4% glucose but no difference under 2% glucose; when the glucose increased to 5 and 6%, the plant size of *agb1*-*2* is smaller than wild type.

**Fig. 4:**
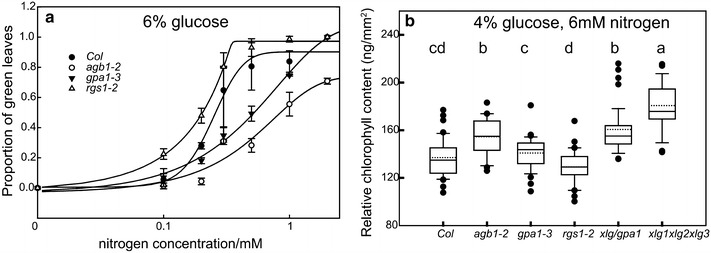
The regulatory pathway of G protein signaling in the C/N sensing evaluated by RGB value. **a** Proportion of green leaves of the G protein mutants in response to nitrogen under 6% glucose. To distinguish from green and not green leaves, a threshold of was established (>15 ng/mm^2^ = *green*) Experiments were repeated twice with 48 seedlings each. The curves were created through global curve fitting (sigmoid equation with four parameters) with SigmaPlot 12.5. **b** The chlorophyll content estimated by RGB value under moderate C/N stress (4% glucose and 6 mM nitrate, n = 40). For the box plot, *solid line* indicates the median and the *dotted line* indicates the mean value. *Different lowercase letters* indicate the significant differences among six genotypes (*p* < 0.05)

We estimated the chlorophyll content in the G protein mutants. The optimal concentration for the growth of *Arabidopsis* grown on agar is 1–2% glucose (Additional file 12: S12B). The G protein complex is involve in glucose sensing [25] and AtRGS1 is a component of a glucose sensor [11, 12, 18]. Previous studies showed that the *rgs1*-2 null mutant is tolerant to high glucose levels [7], however that report used a semi-quantitative method, the so-called “green-seedling” assay. In order to compare the present quantitative results to the published semi-quantitative results, we established a threshold value of 15 ng/mm^2^ to distinguish yellow seedlings from green seedlings (Additional file 12: S12C; the threshold value was from inner fence of *agb1*-*2* mutants’ box plot). Taking *rgs1*-2 mutants as an example (Fig. 4a), the proportion of green leaves was fourfold greater than wild type when the nitrogen concentration was 0.1 mM (0.22 and 0.05 for *rgs1*-2 and Col, respectively). When the nitrogen concentration was increased to 1 mM, the proportion of green leaves increased to 0.98 while the wild type was 0.83. Consistent with the results of Chen and Jones [7], the *rgs1*-2 mutants showed more than 90% green seedlings versus wild type seedlings which scored less than 40%. In addition, as previously observed, the *agb1*-2 mutants contain less chlorophyll than wild type at high glucose and low nitrogen growth conditions.

We also examined the chlorophyll content of the G protein mutants under moderate glucose and high nitrogen (4% glucose, 6 mM nitrogen) stress. Interestingly, as shown in Fig. 4b, the chlorophyll content of the xlg and agb1 mutants were significantly higher compared to the wild type under slight stress, whereas the *rgs1*-2 mutant was not significantly different from wild type.

## Conclusion

This method offer a non-destructive, sensitive, and quantitative way to estimate the chlorophyll content of *Arabidopsis* small seedlings grown on agar plates, and it is an effective way to evaluate the growth condition of the plants. This method provides a quantitative alternative to the qualitative ‘green’ versus not ‘green’ seedling assay [26, 27]. The use of the X-rite ColorChecker chart eliminates differences between cameras and environment, however, caution should be applied in comparing images from highly different environments. Although this method is dependent on the area of the seedlings, rather than the volume, it still provides a good estimate of the relative chlorophyll content for the *Arabidopsis*.

## Additional files

**Additional file 1: S1.** Chlorophyll_imager.java. This is the .java compiler for creating the .class file plug-in for imageJ to be used for the chlorophyll reads.

**Additional file 2: S2.** Chlorophyll_imager.class. This is the plug-in for imageJ to be used for the chlorophyll reads.

**Additional file 3: S3.** RGB_Measure.java This is the .java compiler for creating the .class file plug-in for imageJ to be used for the the RGB reads.

**Additional file 4: S4.** RGB_Measure.class. This is the plug-in for imageJ used for the RGB reads.

**Additional file 5: S5.** The chlorophyll content variation due to the position of the subject.

**Additional file 6: S6.** Cleanbackground.jpg. An example of the cleaned background JPG image.

**Additional file 7: S7.** Greyscaleimage.jpg. The output greyscale images created by imageJ indicated the chlorophyll content.

**Additional file 8: S8.** The chlorophyll content estimated by RGB value for Additional file 5: S5.

**Additional file 9: S9.** EXAMPLE.xlsx. An example of the data set format for the least square equation.

**Additional file 10: S10.** Leastsquareequation.m Script written for Matlab to apply the least square equation.

**Additional file 11: S11.** Raw data for fitting. The original data used for the chlorophyll content calculation with chemical extraction and RGB value, including 4 independent experiment data of the plates grown seedlings and 1 experiment for the plants grown in the soil.

**Additional file 12: S12.** The effect of different C/N ratios on *Arabidopsis* seedlings growth and chlorophyll content. **a** Box plot shows plant sizes in response to C/N ratio. Solid line indicates the median and the dotted line indicates the mean value. **b** The plant leave area of 1 mM nitrogen under different glucose concentrations. **c** The chlorophyll content of G protein mutants under 0.2 mM nitrogen and 6% glucose.

**Additional file 13: S13.** The effect of different C/N ratios on G protein mutants. Growth condition and treatments are as described for Fig. 3. Data analysis was performed by software SAS8.0. Single factor analysis (n = 48). Different capital letters indicate the similarity groups among four genotypes (*p* < 0.05) and lowercase letters indicated the similarity groups among five nitrogen treatments (*p* < 0.05).

## Abbreviations

RGB: red, green and blue channel of the color
r, g, b: the values of the red, green and blue channels
r′, g′, b′: the correction value of the red, green and blue channels
C/N: carbon to nitrogen ratio
MS medium: Murashige and Skoog medium
MES: 2-(*N*-morpholino) ethanesulfonic acid
