# Supplementary figures and images for: A nondestructive method to estimate the chlorophyll content of Arabidopsis seedlings
Source: Plant Methods. 2017 Apr 14;13:26. doi: 10.1186/s13007-017-0174-6 (PMC5391588; doi:10.1186/s13007-017-0174-6)

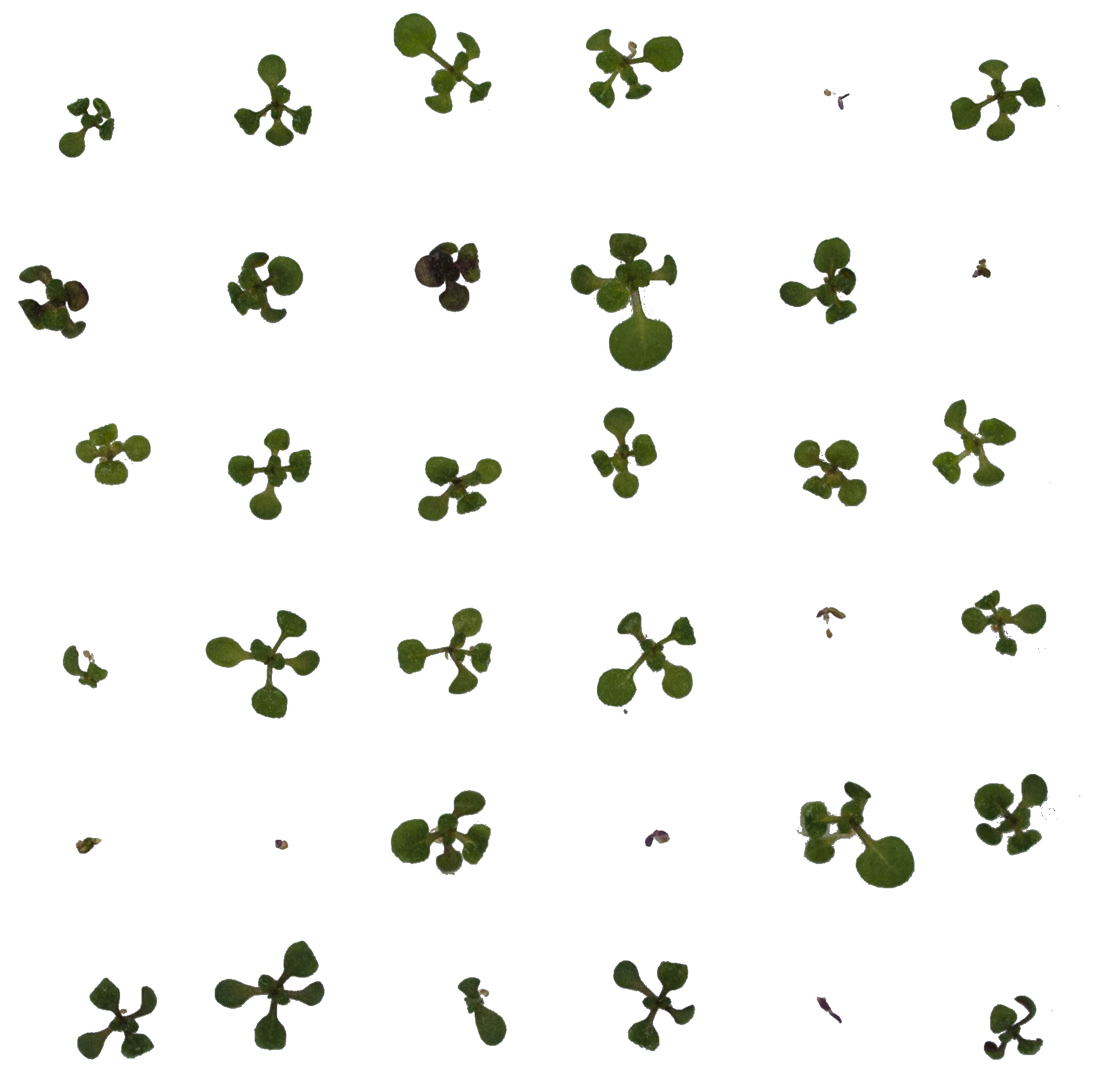

Supplement: Supplementary file 6 — Additional file 6: S6. Cleanbackground.jpg. An example of the cleaned background JPG image. [file 13007_2017_174_MOESM6_ESM.jpg]

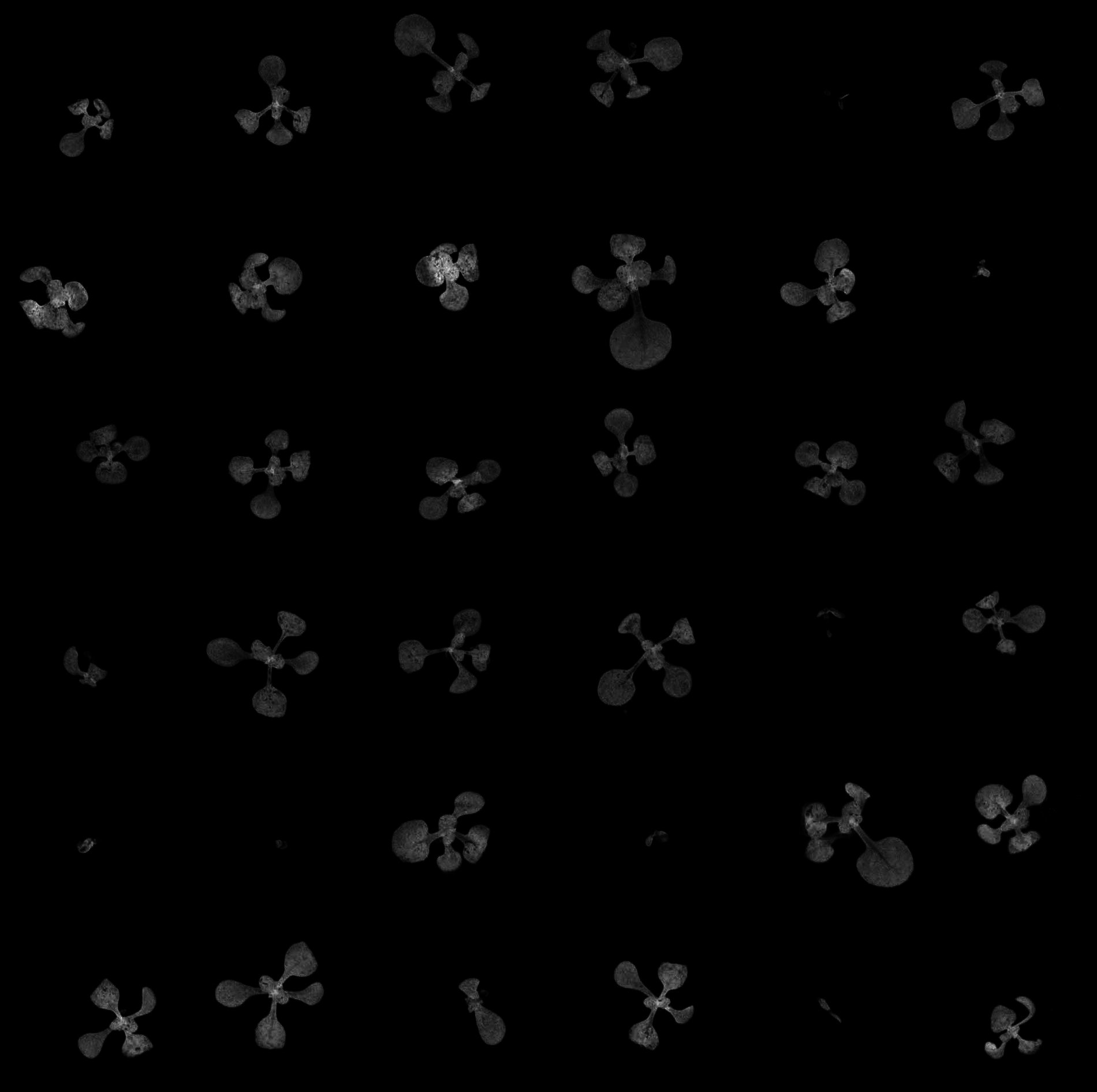

Supplement: Supplementary file 7 — Additional file 7: S7. Greyscaleimage.jpg. The output greyscale images created by imageJ indicated the chlorophyll content. [file 13007_2017_174_MOESM7_ESM.jpg]

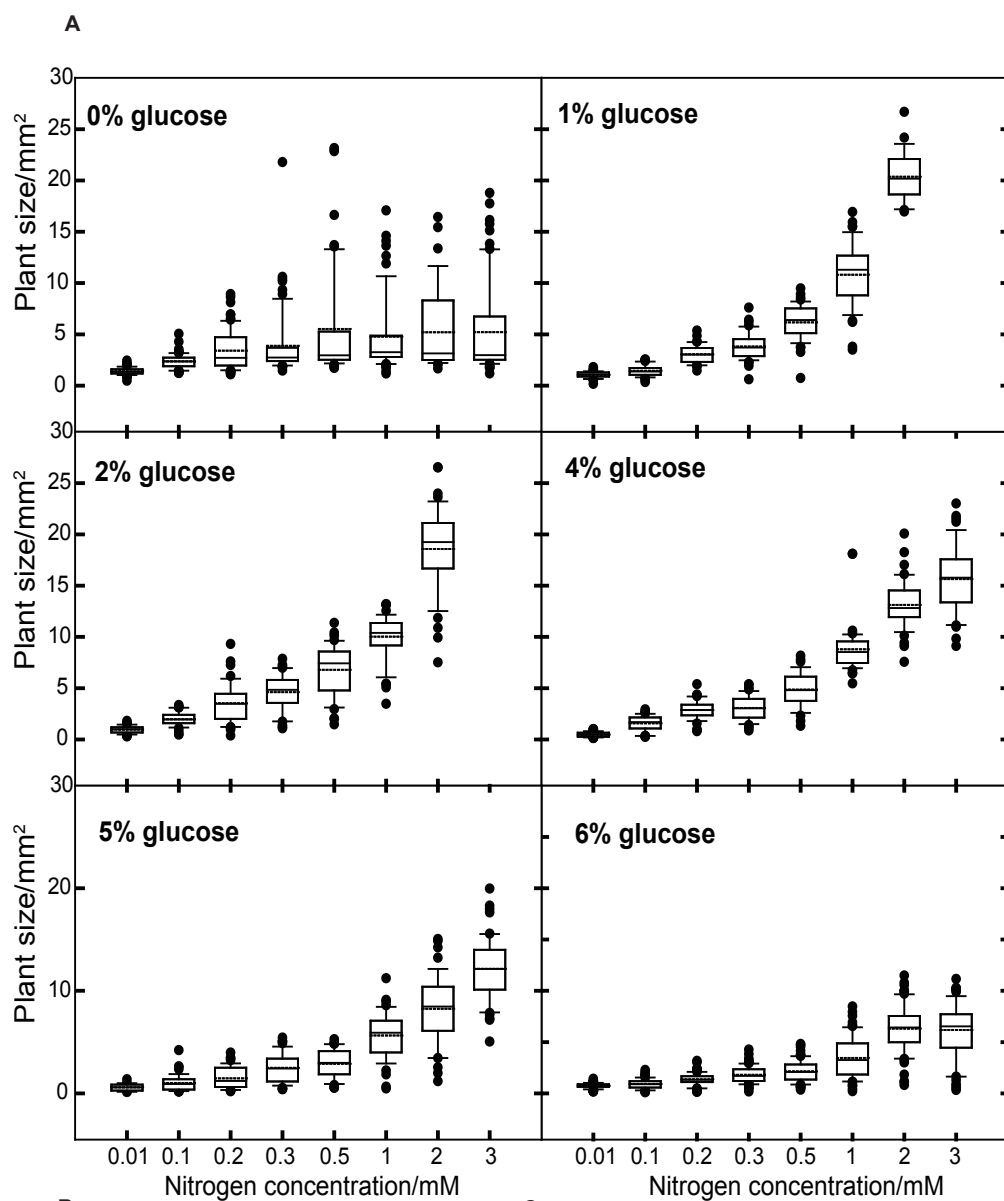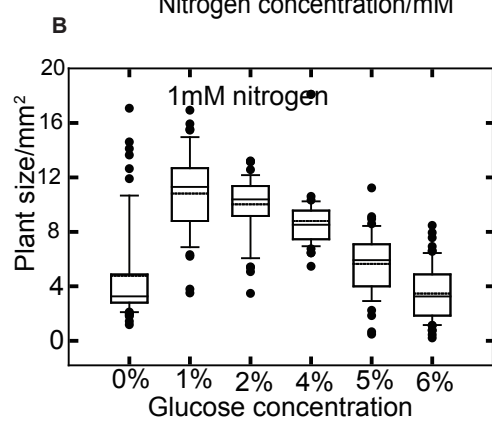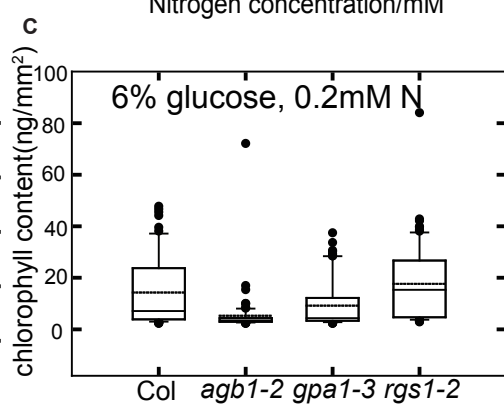

Supplement: Supplementary file 12 — Additional file 12: S12. The effect of different C/N ratios on Arabidopsis seedlings growth and chlorophyll content. a Box plot shows plant sizes in response to C/N ratio. Solid line indicates the median and the dotted line indicates the mean value. b The plant leave area of 1 mM nitrogen under different glucose concentrations. c The chlorophyll content of G protein mutants under 0.2 mM nitrogen and 6% glucose. [file 13007_2017_174_MOESM12_ESM.pdf]
