# Supplementary material for: A nondestructive method to estimate the chlorophyll content of Arabidopsis seedlings
Source: Plant Methods. 2017 Apr 14;13:26. doi: 10.1186/s13007-017-0174-6 (PMC5391588; doi:10.1186/s13007-017-0174-6)
Supplement: Supplementary file 8 — Additional file 8: S8. The chlorophyll content estimated by RGB value for Additional file 5: S5. [file 13007_2017_174_MOESM8_ESM.pdf]

Table S7. The chlorophyll content estimated by RGB value.

	row	column	chlorophyll content(ng/mm2)
1	1	1	159.522
2	1	2	155.212
3	1	3	152.971
4	1	4	149.466
5	1	5	33.439
6	1	6	151.412
7	2	1	137.406
8	2	2	161.738
9	2	3	115.086
10	2	4	157.351
11	2	5	166.794
12	2	6	62.181
13	3	1	109.046
14	3	2	154.116
15	3	3	139.313
16	3	4	134.92
17	3	5	125.919
18	3	6	127.459
19	4	1	114.466
20	4	2	149.465
21	4	3	125.738
22	4	4	148.235
23	4	5	41.628
24	4	6	134.199
25	5	1	61.765
26	5	2	31.744
27	5	3	148.452
28	5	4	40.68
29	5	5	152.128
30	5	6	165.127
31	6	1	158.239
32	6	2	164.719
33	6	3	141.529
34	6	4	156.279
35	6	5	46.432
36	6	6	137.508
