# Supplementary material for: A nondestructive method to estimate the chlorophyll content of Arabidopsis seedlings
Source: Plant Methods. 2017 Apr 14;13:26. doi: 10.1186/s13007-017-0174-6 (PMC5391588; doi:10.1186/s13007-017-0174-6)
Supplement: Supplementary file 13 — Additional file 13: S13. The effect of different C/N ratios on G protein mutants. Growth condition and treatments are as described for Fig. 3. Data analysis was performed by software SAS8.0. Single factor analysis (n = 48). Different capital letters indicate the similarity groups among four genotypes (p < 0.05) and lowercase letters indicated the similarity groups among five nitrogen treatments (p < 0.05). [file 13007_2017_174_MOESM13_ESM.pdf]

Table S13. The plant size (mm^2) of G protein mutants in response to C/N ratio.

Glucose(w/v)	Nitrogen	0mM	0.1mM	0.2mM	0.3mM	0.5mM	1mM	2mM
0%	*Col*	1.38 A[1] e[2]	2.33 A de	3.33 AB cd	3.83 B bc	5.46 A a	4.75 AB ab	5.12 B a
	agb1-2	1.45 A d	2.48 A cd	2.39 C cd	3.57 B bc	4.60 A ab	4.67 B ab	5.67 B a
	gpa1-3	1.37 A e	2.47 A de	2.86 BC d	3.65 B cd	5.20 A b	4.24 AB bc	9.52 A a
	rgs1-2	1.17 B e	2.59 A de	3.48 A cd	6.03 A b	6.06 A b	5.82 A b	9.37 A a
1%	*Col*	1.15 B e	1.45 C de	3.37 B d	3.81 B d	6.66 A c	10.91 B b	24.76 B a
	agb1-2	1.13 B f	2.12 A de	3.66 AB e	4.92 B d	6.66 A c	11.52 A b	23.67 A a
	gpa1-3	2.21 A e	1.67 BC de	4.81 AB d	5.87 B d	8.30 A c	14.19 B b	21.78 B a
	rgs1-2	1.85 AB f	1.86 B de	4.08 A e	5.19 A cd	7.39 A c	12.27 AB b	21.38 B a
2%	*Col*	0.96 B g	1.97 A e	3.53 A e	4.62 B d	6.79 AB c	10.03 B b	18.58 AB a
	agb1-2	0.88 B f	2.11 A f	2.77 A e	4.31 B d	6.40 B c	9.08 B b	18.01 B a
	gpa1-3	0.92 B f	2.05 A e	3.08 A e	4.34 B d	7.50 A c	12.25 A b	20.21 A a
	rgs1-2	1.61 A f	2.24 A ef	3.32 A de	4.45 A cd	4.67 C c	9.19 B b	17.63 B a
4%	*Col*	0.47 B f	1.58 B e	2.87 BC d	3.07 B d	4.86 C c	8.80 B b	13.13 B a
	agb1-2	0.51 B f	1.92 A e	2.88 C d	2.97 B d	6.23 A c	8.50 B b	16.62 A a
	gpa1-3	0.62 A g	1.61 B f	3.00 B e	3.98 A d	5.36 BC c	10.34 A b	17.64 A a
	rgs1-2	0.51 B f	1.54 B e	3.15 A d	3.22 B d	5.86 AB c	8.25 B b	14.27 B a
5%	*Col*	0.59 AB e	1.01 AB e	1.47 B cd	2.50 A cd	2.88 B c	5.65 B b	8.25 C a
	agb1-2	0.49 B c	0.80 B c	1.07 C c	1.84 B c	1.92 C c	4.14 C b	7.60 C a
	gpa1-3	0.67 A d	1.24 A d	1.71 B cd	2.93 A cd	4.10 A bc	6.32 AB b	12.10 B a
	rgs1-2	0.63 AB f	1.10 A ef	2.15 A de	2.70 A d	4.44 A c	6.97 A b	10.42 A a
6%	*Col*	0.74 AB f	0.92 A ef	1.37 B de	1.81 A cd	2.15 B c	3.46 B b	6.32 A a
	agb1-2	0.77 A c	0.87 A c	1.02 C c	1.26 B c	1.32 C c	2.30 C b	2.93 B a
	gpa1-3	0.76 A d	0.98 A de	1.29 B cde	1.67 A cd	1.80 B c	3.33 B b	6.26 A a
	rgs1-2	0.66 B e	0.97 A e	1.61 A d	1.84 A d	2.79 A c	4.36 A b	6.31 A a

Note: Different lowercase letters[1] indicated significant differences among nitrogen treatment and different capital letters[2] indicated significant difference among glucose treatment, n=12
